# Tissue Reaction Elicited by Neosealer Flo, AH 26, and CC Sealer in Rats: Tissue Reaction of CC Sealer

**DOI:** 10.31661/gmj.v13iSP1.3599

**Published:** 2024-12-08

**Authors:** Elham Mahdavisefat, Kiumars Nazarimoghaddam, Reza Sedaghat, Hossein Labbaf, Jalil Modaresi, Mohsen Khalili, Zahra Jafari

**Affiliations:** ^1^ Department of Endodontics, School of Dentistry, Shahed University, Tehran, Iran; ^2^ Department of Anatomy and Pathology, Immunoregulation Research Center, School of Medicine, Shahed University, Tehran, Iran; ^3^ Department of Endodontics, School of Dentistry, Shahid Sadoughi University of Medical Sciences, Yazd, Iran; ^4^ Neurophysiology Research Center, School of Medicine, Shahed University, Tehran, Iran

**Keywords:** Animal, Canals Sealer, Biocompatible Materials, Calcium Silicate

## Abstract

**Background:**

Since root canal sealers are in contact with periradicular tissues, biocompatibility is one of their most important features. There is no available study about the biocompatibility of NSF and CC Sealer that are newly made bioceramic-based sealers. This study aimed to compare the tissue reaction elicited by NeoSealer Flo (NSF), AH26, and ColdCeramic Sealer (CC sealer) in rats.

**Materials and Methods:**

The sealers were mixed and applied in molds to fabricate sealer discs, which were then implanted in the subcutaneous tissue of the backs of 30 healthy adult Albino Wistar rats. Each rat received three sealer discs and the fourth incision site remained empty as a control group. After 7, 30, and 90 days, the rats were sacrificed. Biopsy samples were evaluated regarding the extent and severity of inflammation, angiogenesis, fibroplasia, and infiltration. Data were analyzed in SPSS software using Kruskal-Wallis and Mann-Whitney U tests

**Results:**

Tissue reaction to NSF was generally severe and increased up to day 30, but slightly decreased at three months, although it was still severe, and significantly greater than the tissue reaction to other sealer types. After one month, all rats in the NSF group showed foreign body reaction and giant cells around sealer particles; while, foreign body reaction was not seen in other groups. Tissue reaction to CC Sealer and AH26 was not significantly different at any point in time (P0.05) and was the highest on day seven and then decreased up to month three.

**Conclusion:**

According to the present results, the CC Sealer appears to be a biocompatible material; however, NSF showed higher severity and extent of inflammation and triggered higher tissue reaction.

## Introduction

Successful endodontic treatment depends on adequate instrumentation, disinfection, and obturation of the root canal system [1]. In obturation with gutta-percha, voids are filled with sealers to seal the root canal [2].Grossman in 1988 described the characteristics of an ideal sealer. Accordingly, an ideal sealer must be biocompatible [1]. In contact with the periradicular tissue, sealers release various substances and cause different reactions [3]. Since root canal sealers are in contact with periradicular tissues, biocompatibility is one of their most important features [4]. By the advances in science and technology, the outcome of endodontic treatment has profoundly improved [5]. However, most sealers show varying degrees of cytotoxicity and tissue reactions, that affect the efficacy of treatment [6].

Biocompatibility refers to not causing an adverse reaction in tissue contact. It can be determined by looking for cellular infiltration or vascular changes and assessing the severity of the inflammatory response [7].

ColdCeramic Sealer (CC Sealer) is a newly made bioceramic-based sealer. It is a powder/liquid system. Its base is ColdCeramic, a bioceramic and hydrophilic cement used for perforation managment, vital pulp therapy and apicoectomy. Its chemical composition contains Calcium oxide, silicon dioxide, sulfur oxide and barium oxide as a radiopacifier. Its microleakage, biocompatibility, and alkaline pH are similar to those of mineral trioxide aggregate (MTA). It is not water soluble and gains some weight following immersion in water [8].Assessment of tissue reaction shows that both MTA and ColdCeramic are well tolerated by the tissues [9].

NeoSealer Flo (NSF) (Neosealer Flo Avalon Biomed, USA) is a premixed bioceramic-based sealer consisting of tricalcium silicate, dicalcium silicate, calcium aluminate, calcium aluminum oxide (grossite), tricalcium aluminate, tantalite as radiopacifier and Traces of calcium sulfate (<1%) [10]. This study aimed to compare tissue reaction to AH26, CC Sealer, and NSF in rats. NSF was chosen because it has recently been introduced as a bioceramic sealer in the market and many favorable properties such as resin-free, biocompatible, bioactive, and promoting the forming of hydroxyapatite on the dentine have been stated for it [11], But there is no available study about the biocompatibility of NSF. CC Sealer also is a newly made bioceramic sealer and there is no available study about its biocompatibility too. AH26 (Dentsply, DeTrey, Konstanz, Germany) is an epoxy-resin based sealer and has extensive applications for root canal obturation because of its good properties such as tissue tolerance, slow setting time, solubility in solvents, etc [12].

## Materials and Methods

**Figure-1:**
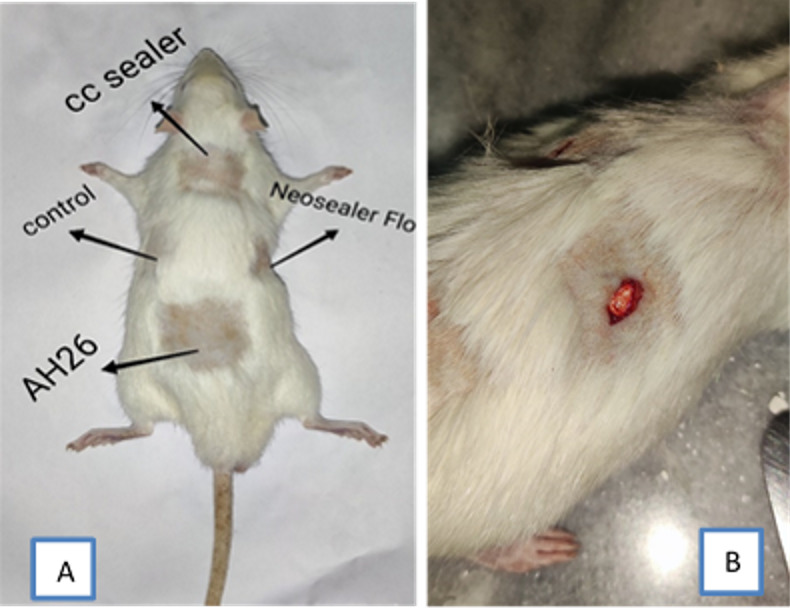
Figure 1. The back of the rat (A) Surgical sites of sealers (B)surgical incision and implanted sealer

This animal study was conducted on 30 healthy adult Albino Wistar rats weighing 175 to 200 g in the Animal laboratory of Shahed university. The study method had been approved by the ethics committee of Shahed University of Medical Sciences (IR.SHAHED.REC.1401.115).

### Sample Size

The sample size was calculated to be 10 in each sealer group assuming d=0.05, confidence interval of 0.95, and Z=1.96 [7].

### Intervention

The rats were kept under standard conditions with the same diet for one week before the experiment for acclimation.

The sealers were mixed according to the manufacturer’s instructions and applied in molds measuring 5 mm in diameter and 2 mm in height by using a spatula. The surface of the molds was covered with a moist gauze to allow them to set in the presence of moisture. The discs were stored at room temperature for 48 hours before implantation.

On the day of surgery, the rats were generally anesthetized by intraperitoneal injection of ketamine (Panpharma GmbH, Germany) and xylazine (Alfasan, Netherlands). The recommended dose of ketamine/ xylazine for surgical anesthesia in rats is 100 mg/kg and 10 mg/kg, respectively [13]. The back of the rats was shaved and disinfected with alcoholl %70. Next, four incisions with 10 mm length and 5 mm depth were made with a number 15 scalpel blade (moris) in the subcutaneous tissue of the back each rat. AH26 sealer, CC Sealer, and NSF discs were implanted at the three incision sites, while the fourth incision site remained empty as the control group (Figure-1). The incisions were then sutured with 4.0 nylon thread (monofilament polyamide non-resorbable).

After 7, 30, and 90 days, the rats were sacrificed in a CO2 gas chamber [14]. To euthanize the rats, they were placed in a 28-L CO2 chamber. The chamber’s door was closed and CO2 gas entered the chamber at a flow rate of 6.5 L/minute for five minutes and continued for one more minute after cessation of respiration. Next, the incision sites were biopsied and the biopsy specimens were fixed with 10% buffered formalin for one week. Then, they were placed in paraffin blocks. A rotary microtome were used to Serial sections. They were then stained with hematoxylin and eosin.

The following parameters were then evaluated and scored by a blinded histopathologist, under a microscope. (Labomed, USA). Although these criteria are conventional and are used in different ways. Because all groups are evaluated and graded with the same scale, it can be reliable [15]:

Extent of Inflammation:

Score 0: No inflammatory cell infiltration

Score 1: Inflammatory cells in one or two microscopic fields

Score 2: Inflammatory cells in three to five microscopic fields

Score 3: Inflammatory cells in more than five microscopic fields

Severity of inflammation:

Score 0: No inflammatory cell

Score 1: Large empty spaces between the inflammatory cells, and low cellular density

Score 2: Small empty spaces between the inflammatory cells

Score 3: No empty space between the inflammatory cellsAngiogenesis:

Score 0: No blood vessels

Score 1: one to five blood vessels per microscopic field

Score 2: six to 10 blood vessels per microscopic field

Score 3: more than 10 blood vessels per microscopic field

Fibroplasia (formation of fibroblasts and extracellular matrix):

Score 0: No fibroblasts

Score 1: High number of fibroblasts, high amounts of collagen, and presence of fine fibers per each microscopic field

Score 2: Moderate number of fibroblasts, moderate amount of relatively thick collagen fibers

Score 3: Small number of fibroblasts, thick collagen fibers

Inflammatory cell infiltration:

Foreign body reaction

Mixed with polymorphonuclear dominance (PMD)

Mixed with mononuclear dominance

Mononuclear cell infiltration

None

### Statistical Analysis

Data were analyzed using SPSS version 25 (SPSS Inc., IL, USA). The normality of data distribution was evaluated by The Shapiro-Wilk test and showed that it wasn’t normally distributed. Thus, the Kruskal-Wallis test was used employed to comparing the severity of inflammation, in both the case and control groups for each interval (7, 30, and 90). If results were significant, the Mann-Whitney U test was employed to compare the pairwise groups.

## Results

**Table:** Table1. Mean Rank of Variables in the Four Groups (n=10)

		**Day 7**	**Day 30**	**Day 90**
	Control	7.25	6.5	8.5
**Extent of inflammation**	NSF	32.15	35.5	35.5
	CC Sealer	23.75	18.2	19
	AH26	18.85	21.8	19
	Control	9.25	13	14
**Severity of inflammation**	NSF	33.1	35.5	35.5
	CC Sealer	23.2	16	15.5
	AH26	16.45	17.5	17
	Control	13.55	9.25	9.5
**Angiogenesis**	NSF	21.75	29.4	33.5
	CC Sealer	27.3	18.35	20.85
	AH26	19.4	25	18.15
	Control	18.7	32.4	23
**Fibroplasia**	NSF	18.1	11.8	8.6
	CC Sealer	18.7	19.8	24.55
	AH26	26.5	18	25.85

### Day 7

Table-1 shows the mean rank of different variables in the four groups at seven days. On day seven, the control group showed the lowest severity and extent of inflammation, and angiogenesis. The NSF group showed the highest severity and extent of inflammation. The CC Sealer group showed the highest angiogenesis. Kruskal-Wallis showed that the difference in the extent (P<0.001) and severity (P<0.001) of inflammation and angiogenesis (P=0.047) was significant. However, the difference in fibroplasia (P=0.136) was not significant. Mann-Whitney U test showed significant differences between all groups (P<0.05) except between AH26 and CC sealer (P>0.05) regarding the extent and severity of inflammation. Also, significant differences were found between the control group and CC Sealer regarding angiogenesis on day seven (P<0.05).

### Day 30

Table-1 shows the mean rank of different variables in the four groups at 30 days. On day 30, the control group showed the lowest severity and extent of inflammation, and angiogenesis, and the highest rate of fibroplasia. The NSF group showed the highest severity and extent of inflammation. Kruskal-Wallis showed significant differences in the extent (P<0.001), and severity (P<0.001) of inflammation, angiogenesis (P<0.001), and fibroplasia (P<0.001) among the groups. Mann-Whitney U test showed the differences between all groups were significant (P<0.05) except between AH26 and CC sealer (P>0.05) regarding the extent of inflammation. Also, significant differences were found between NSF and all other groups regarding the severity of inflammation (P<0.05). Pairwise comparisons of the sealer groups test were also noted between all groups (P<0.05) except between AH26 and NSF, and AH26 and CC sealer groups (P>0.05) regarding angiogenesis. The differences among all groups were also found to be significant (P<0.05) except between AH26 and CC sealer (P>0.05) regarding fibroplasia on day 30.

### Day 90

Table-1 shows the mean rank of different variables in the four groups at 90 days. On day 90, the control group showed the lowest extent and severity of inflammation, and angiogenesis in the control group. The NSF group showed the highest extent and severity of inflammation, and angiogenesis, and the lowest rate of fibroplasia. Kruskal-Wallis revealed significant differences in the extent of inflammation (P<0.001), severity of inflammation (P<0.001), angiogenesis (P<0.001), and fibroplasia (P<0.001). Mann-Whitney U test showed the same results as those reported for day 30 regarding the extent and severity of inflammation, and significant differences were found between all groups (P<0.05) except AH26 and CC sealer (P>0.05) regarding angiogenesis. Also, significant differences were noted between NSF and all other groups regarding fibroplasia on day 90 (P<0.05).

### Comparison of Tissue Reaction

**Figure-2:**
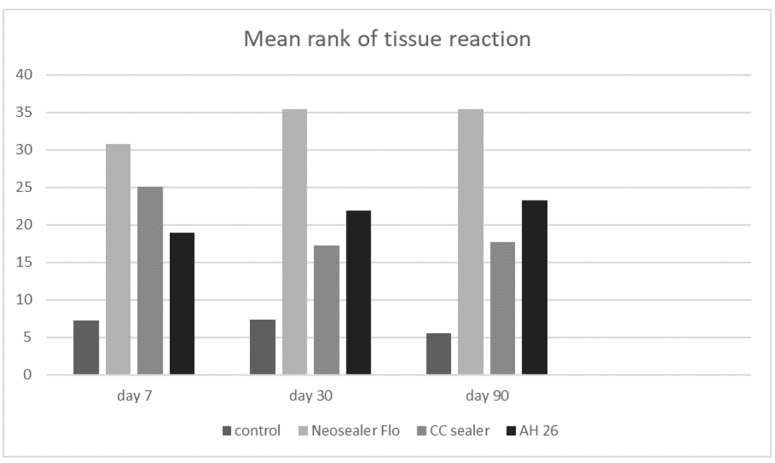
Figure 2. Mean rank of tissue reaction in the four groups at different time points

Tissue reaction is a combination of the Extent of inflammation, the severity of inflammation, and angiogenesis criteria. Significant differences were noted in tissue reaction among the four groups at 7 days, 30 days, and 90 days (P<0.001 for all). The lowest tissue reaction was noted in the control group and the highest in the NSF group (Figure-2). On day seven, the tissue reaction was the highest in the NSF, followed by the CC Sealer, and AH26 group. The differences between AH26 and CC Sealer, and also CC Sealer and NSF were not significant. However, the difference between AH26 and NSF was significant. On day 30, tissue reaction was the highest in the NSF, followed by AH26 and then CC Sealer group. The difference between AH26 and CC Sealer was not significant (P>0.05). However, the differences between AH26 and CC Sealer, and AH26 and NSF were significant. On day 90, tissue reaction was the highest in the NSF, and equal in AH26 and CC Sealer groups. AH26 and CC Sealer had a significant difference with NSF in this regard.

### Infiltration

Table-2 shows the frequency of infiltration degrees in different sealer groups at various time points.

**Figure-3:**
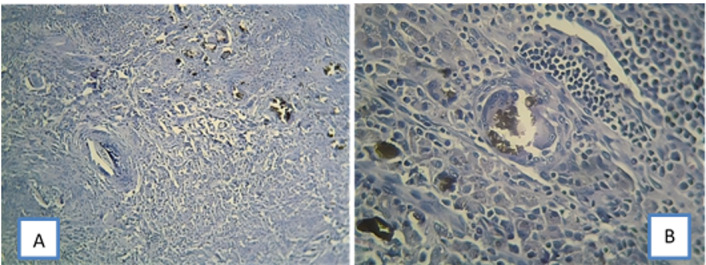
Figure 3. (A) subcutaneous tissue reaction to NSF on day 90. Foreign body reaction against the implanted material (brown material) can be seen. x100 magnification. (B) subcutaneous tissue reaction to NSF on day 90. Infiltration of mononuclear cells, plasma cells, and giant cells, and foreign body reaction to brown material can be seen. x400 magnification, H & E staining.

**Figure-4:**
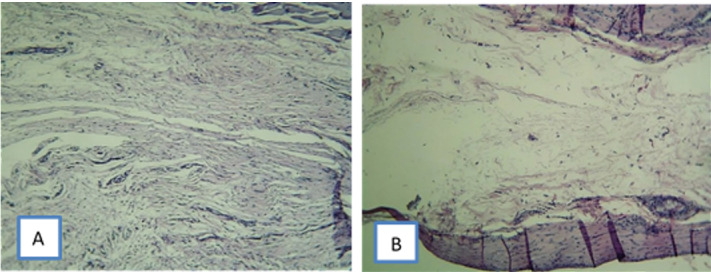
Figure 4. Tissue reactions on day 90: (A) subcutaneous tissue reaction to CC Sealer; No sign of inflammation can be seen. Collagen fibers are formed, and fibroblasts are mature. The connective tissue has low cellularity. B) subcutaneous tissue reaction to AH26. A capsule can be seen around the implanted material. CC Sealer group shows higher tissue maturity and lower angiogenesis than AH26 group. x100 magnification, H & E staining

Figures-3 and -4 show micrographs of tissue reactions in different sealer groups.

## Discussion

**Table:** Table2. Frequency of Infiltration Degrees in Different Sealer Groups at Different Time Points

**Time**	**Infiltration degree**	**Control**	**NSF**	**CC Sealer**	**AH26**
**7 days**	Foreign body reaction Mixed with PMD Mixed with mononuclear dominance Mononuclear cell infiltration None Total	0 0 0 1(10%) 9(9%) 0 10 (100‌%)	0 1 (10‌%) 2 (20‌%) 5 (50‌%) 2 (20‌%) 0 10 (100‌%)	0 0 0 0 10 (100‌%) 0 10 (100%)	0 0 0 3(30%) 7(70%) 0 10 (100‌%)
**30 days**	Foreign body reaction Mixed with PMD Mixed with mononuclear dominance Mononuclear cell infiltration None Total	0 0 0 0 1(10%) 9(90%) 10 (100‌%)	10(100%) 0 0 0 0 0 10 (100‌%)	0 0 0 0 3(30%) 7(70%) 10 (100‌%)	0 0 0 0 4(40%) 6(60%) 10 (100%)
**90 days**	Foreign body reaction Mixed with PMD Mixed with mononuclear dominance Mononuclear cell infiltration None Total	0 0 0 0 0 10 (100‌%) 10 (100‌%)	10(100%) 0 0 0 0 0 10 (100%)	0 0 0 0 0 10 (100‌%) 10 (100‌%)	0 0 0 0 2(20%) 8(80%) 10 (100‌%)

In endodontic treatment, sealers that have the least cytotoxic effects on the periapical tissue and are well tolerated by the adjacent tissues are preferred [14].

This study was conducted to compare tissue reaction elicited by AH26, NSF, and a new bioceramic-based sealer called CC Sealer in rats. This study was the first to assess the tissue reaction elicited by CC Sealer and NSF and compare it with AH26, which has widespread applications for root canal obturation and has been tissue-tolerated for many years [14].

CC Sealer and NSF are calcium silicate-based sealers, and primary inflammatory reactions are expected [8]. The recruitment of inflammatory cells is usually associated with alkaline pH provided by bioceramic materials in contact with tissue and, the release of calcium and other substances as dispersing agents [16].

Tavares et al. [17] compared connective tissue reactions to MTA Fillapex, EndoFill zinc oxide sealer, and AH Plus in Wistar rats and reported that none of the sealers produced optimal tissue reactions. Their results aligned with the findings presented in this study. The results indicated varying levels of inflammation in all sealer groups, significantly exceeding those observed in the control group. Mild inflammatory reactions in the control group were probably due to surgical trauma. Over time, inflammation significantly decreased in the control group.

Ashraf et al. [18] reported a reduction in severity and percentage of inflammation over time in all sealer groups; although this reduction was not significant while Kohsar et al. [19] evaluated submucosal tissue reactions in rats elicited by Adseal and SureSeal Root and showed that the severity of inflammation decreased in both the test and control groups at 30 and 60 days. In the present study, the severity and extent of inflammation gradually increased in the NSF group while in the AH26 and CC Sealer groups, the extent and severity of inflammation significantly decreased during these three months.The present study showed that tissue reaction to the CC Sealer group was comparable to that in the AH26 group with no significant difference in any parameter at any time points.

However, Tissue reaction to NSF was generally severe and increased up to 30 days, but slightly decreased at three months, although it was still severe, and significantly greater than the tissue reaction to other sealer types. After the first month, all NSF samples showed foreign body reaction and giant cells around sealer particles while foreign body reaction was observed in any of the other groups.

We couldn’t find any other studies on the biocompatibility of NeoSealer Flo but one study evaluated the inflammatory reaction and mineralization activity of NeoPUTTY, comparing it with Bio-C Repair and MTA Repair HP and all periods, NeoPutty specimens contained the highest values of Inflammatory cells [16]. Another study compared the biocompatibility and bioactive potential of NeoMTA Plus with that of MTA Fillapex. At seven days, the capsules surrounding both NeoMTA Plus and MTA Fillapex contained more inflammatory cells and IL-6-immunostained cells than the control group (CG). However, after 60 days, the difference in the number of inflammatory cells between the two sealers was insignificant. Nevertheless, a higher number of IL-6-immunostained cells was observed in the MTA Fillapex group [20].

Angiogenesis at one and three months was greater in the NSF group than in other sealer groups in the present study. Generally, angiogenesis starts on day three, reaches its maximum level on day five, and then subsides [15]. Thus, angiogenesis in the first week is normal and should be decreased in the second week. AH26, CC Sealer, and the control group followed this trend; however, angiogenesis reached its peak at one month in the NSF group, which indicates an inflammatory reaction and unfavorable tissue reaction. Greater fibroplasia indicates a better healing process [15].

Fibroplasia was the lowest in the NSF group at 7, 30 and 90 days. The 90-day samples in the CC Sealer group revealed more favorable healing than AH26 with no sign of inflammation. It appears that the initiation of temporary inflammation in response to endodontic calcium silicate materials plays a fundamental role in subsequent healing. The release of inflammatory cytokines such as interleukin-6 and 1 is related to the next phase of healing in the normal healing process [21] but, In the NSF group, foreign body reaction, giant cells, and infiltration of mononuclear cells and plasma cells were evident after the first month (Figure-3, B), probably due to the constituents of this sealer. In bioceramic sealers, calcium aluminate can be used alongside tricalcium silicate cement, with or without calcium sulfate. The combination of these two types of cement can reduce setting time; in addition, the presence of calcium sulfate enhances the strength and controlled expansion of the cement.

The formulation used in NeoSealer Flo consists of a mixture of calcium aluminate and calcium silicate, with an excess of calcium sulfate. However, a significant issue arises concerning biocompatibility. The reaction between aluminate cement and tricalcium silicate depletes the calcium hydroxide produced during the tricalcium silicate reaction, leading to reduced biocompatibility, as evidenced by tests conducted using cell cultures [22].

Modaresi et al. [9] showed that ColdCeramic had higher biocompatibility than MTA in rats over longer courses, and fibrous tissue was noted in all rats in the ColdCeramic group after 30 days, which indicated wound healing. Because of the high initial pH of calcium hydroxide, a greater number of necrotic areas were observed in the ColdCeramic group. Mozayeni et al. [23] evaluated the cytotoxicity of ColdCeramic, MTA, and Intermediate Restorative Material, and indicated that MTA had the lowest cytotoxicity followed by ColdCeramic. Variations in the results of studies can be due to different methodologies and scoring systems used for quantification of processes [24].

The present study highlighted the significance of initial inflammation in the regenerative process, such that at three months, the CC Sealer showed no sign of inflammation; collagen fibers had been well formed and the fibroblasts were mature. The connective tissue had low cellularity and in total, the CC Sealer group showed higher tissue maturity in terms of healing than AH26. (Figure-4)

This study was an animal study. Thus, the results cannot be well generalized to humans. The sealers were implanted in the back of rats, which has differences from the periapical tissue in humans in terms of structure and types of immune cells and blood supply. Because of sufficient blood flow in periapical tissue and its difference from the connective tissue of rats, the sealers would have lower cytotoxicity and different tissue reactions in the periapical tissue of humans. Moreover, the diameter of sealer discs (5 mm) is different from the apical foramen diameter, and the contact area with sealers would be different (the surface area would be approximately 100 times greater than that at the apical foramen). Furthermore, the presence of gutta percha in clinical conditions has not been considered in this study and other previous studies. Thus, clinical trials are required to cast a final judgment regarding the properties of sealers.

## Conclusion

According to the present results, CC Sealer appears to be biocompatible; however, NSF showed higher severity and extent of inflammation and triggered higher tissue reaction.

## Conflict of Interest

There is no conflict of interest in connection with this article.
